# YeATS - a tool suite for analyzing RNA-seq derived transcriptome identifies a highly transcribed putative extensin in heartwood/sapwood transition zone in black walnut

**DOI:** 10.12688/f1000research.6617.2

**Published:** 2015-11-06

**Authors:** Sandeep Chakraborty, Monica Britton, Jill Wegrzyn, Timothy Butterfield, Pedro José Martínez-García, Russell L. Reagan, Basuthkar J. Rao, Charles A. Leslie, Mallikarjuna Aradhaya, David Neale, Keith Woeste, Abhaya M. Dandekar

**Affiliations:** 1 Plant Sciences Department, University of California, Davis, CA, 95616, USA; 2 UC Davis Genome Center Bioinformatics Core Facility, University of California, Davis, CA, 95616, USA; 3 Department of Ecology and Evolutionary Biology, University of Connecticut, Storrs, CT, 06269, USA; 4 Department of Biological Sciences, Tata Institute of Fundamental Research, Homi Bhaba Road, Mumbai, 400, India; 5 USDA Forest Service Hardwood Tree Improvement and Regeneration Center, Purdue University, West Lafayette, IN, 47907, USA

**Keywords:** RNA-seq, transcriptome, open reading frame, extensin, proline-rich proteins, dehydrins, senescence-associated proteins, Computational genomics, Juglans nigra, black walnut, heartwood/sapwood transition zone

## Abstract

The transcriptome provides a functional footprint of the genome by enumerating the molecular components of cells and tissues. The field of transcript discovery has been revolutionized through high-throughput mRNA sequencing (RNA-seq). Here, we present a methodology that replicates and improves existing methodologies, and implements a workflow for error estimation and correction followed by genome annotation and transcript abundance estimation for RNA-seq derived transcriptome sequences (YeATS - Yet Another Tool Suite for analyzing RNA-seq derived transcriptome). A unique feature of YeATS is the upfront determination of the errors in the sequencing or transcript assembly process by analyzing open reading frames of transcripts. YeATS identifies transcripts that have not been merged, result in broken open reading frames or contain long repeats as erroneous transcripts. We present the YeATS workflow using a representative sample of the transcriptome from the tissue at the heartwood/sapwood transition zone in black walnut. A novel feature of the transcriptome that emerged from our analysis was the identification of a highly abundant transcript that had no known homologous genes (GenBank accession: KT023102). The amino acid composition of the longest open reading frame of this gene classifies this as a putative extensin. Also, we corroborated the transcriptional abundance of proline-rich proteins, dehydrins, senescence-associated proteins, and the DNAJ family of chaperone proteins. Thus, YeATS presents a workflow for analyzing RNA-seq data with several innovative features that differentiate it from existing software.

## Introduction

Analysis of the complete set of RNA molecules in a cell, the transcriptome, is critical to understanding the functional aspects of the genome of an organism. Most transcripts get translated into proteins by the ribosome ^1^. Non-translated transcripts (noncoding RNAs) may be alternatively spliced and/or broken into smaller RNAs, the importance of which have only recently been recognized ^2^. Transcriptional levels vary significantly based on environmental cues ^3^, and/or disease ^4^. Quantifying transcriptional levels constitutes an important methodology in current biological research. Traditional methods like RNA:DNA hybridization ^5^ and short sequence-based approaches ^6^ have been supplanted recently by a high-throughput DNA sequencing method - RNA-seq ^7, 8^. Concomitant with the introduction of RNA-seq has been the development of a diverse set of computational methods for analyzing the resultant data ^9– 21^.

In the current work, we present a methodology for analyzing RNA-seq data that has been assembled into transcripts ( **YeATS** - **Ye**t **A**nother **T**ool **S**uite for analyzing RNA-seq derived transcriptome). The process of associating genomic open reading frames (ORF) to a set of transcripts (transcriptome) is the key step in YeATS, enabling identification and correction of specific errors arising from sequencing and/or assembly, a novel feature missing in most known tools. These errors include transcripts that have not been merged, a transcript having broken ORFs and transcripts containing long repeats. Also, YeATS identifies noncoding RNAs by comparison to compiled databases ^22^, transcripts with multiple coding sequences and highly transcribed genes (based on simple normalization of raw counts followed by sorting).

Here, the YeATS workflow is demonstrated using a representative sample of the transcriptome from the tissue at the heartwood/sapwood transition zone in black walnut ( *Juglans nigra* L.). We have identified transcripts that have sequencing and/or assembly errors (~5%). A novel feature that emerged from our analysis was the presence of a highly transcribed gene that had no known homologous counterpart in the entire BLAST database. The amino acid composition of the longest open reading frame of this gene consists of a high percentage of leucine, histidine and valine, and classifies this as a putative extensin ^23^. Given the economic and ecological importance of black walnut timber, characterization of such genes will enhance our understanding of the mechanisms underlying the unique properties associated with the wood of these trees ^24^. The significance of proline-rich proteins ^25^, dehydrins ^26^, senescence-associated proteins ^27^ and DNAJ ^28^ proteins to the formation of heartwood was established through their transcriptional abundance. Finally, based on transcripts that have no known homologs, we have identified noncoding RNAs by comparison with the noncoding RNA database for *Arabidopsis* ^22^. Thus, in the current work, we present a workflow (YeATS) with several novel features absent in most currently available software.

## Methods

### *In silico* methods

The input to YeATS is a set of post assembly transcripts as a fasta file ( *φ _TRS_*). The first step is to identify the set of genes (proteins) encoded by *φ _TRS_*. This is done by associating a proper open reading frame (ORF) to each transcript. This involves a comprehensive automated BLAST run ^29^.

**Figure 1.**
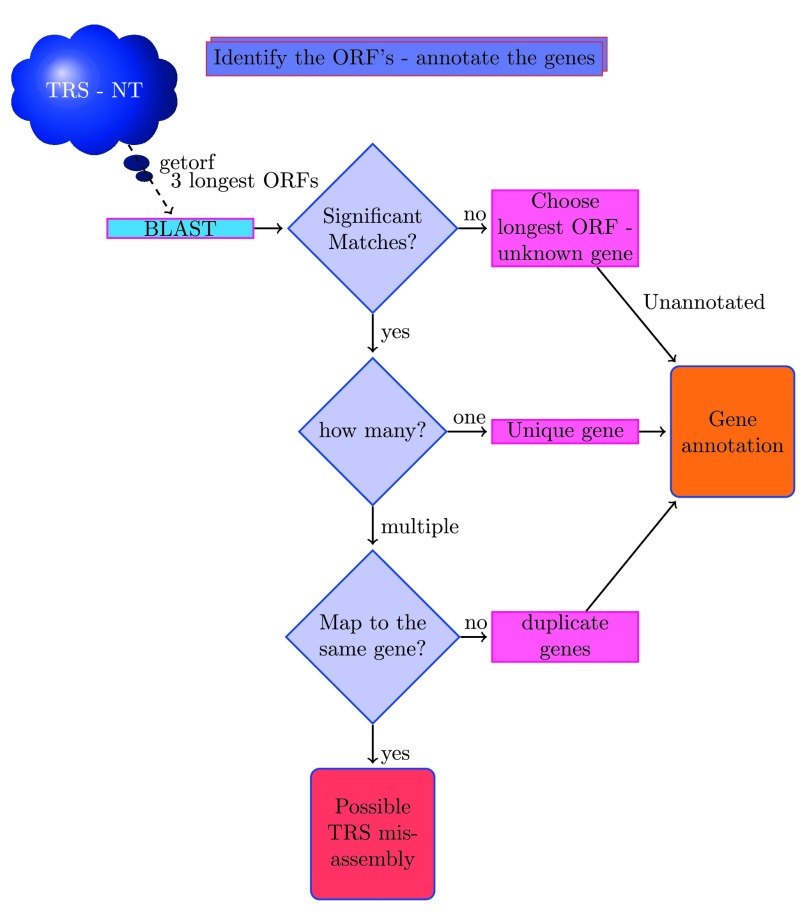
Flowchart for YeATS. For each transcript, the three longest open reading frames (ORF) are obtained using the ‘getorf’, and these were BLAST’ed to the full non-redundant protein sequences (‘nr’) database. Based on the number of significant matches, the transcriptome is partitioned. Unique genes have only one significant match, erroneous transcripts have multiple ORFs matching the same gene, while duplicate genes have multiple distinct matches.

For each transcript in *φ _TRS_*, we generate the three longest ORFs (using the ‘getorf’ utility in the EMBOSS suite ^30^) ( Figure 1). These three ORFs are BLAST’ed to the full non-redundant protein sequences (‘nr’) database. For a given E-value cutoff (1E-12 in the current work), we create four sets

1. Only one ORF is less than the cutoff - the transcript is uniquely annotated.
2. None of the ORFs is less than the cutoff - the transcript has no known homologs.
3. More than one ORF is less than the cutoff.
  (a) The ORFs map to different fragments of the same protein. This points to an error in the sequencing or the assembly, which breaks down the contiguous ORF into two fragments.
  (b) The ORFs map to different proteins - these are instances of a transcript having two valid ORFs. We duplicate the transcript, associating each one to a different protein sequence.

To produce the uniquely annotated set of genes, we ignored entries with the keywords chromosome, hypothetical, unnamed, unknown and uncharacterized, in order to have a functional characteristic in the annotation, provided the final annotated entry has low E-value. Also, apart from comparing E-values, we also compare the BLAST score, choosing an ORF as unique if its BLAST score was more than twice any other BLAST score, even if other scores satisfied the E-value criteria.

**Figure S1.**
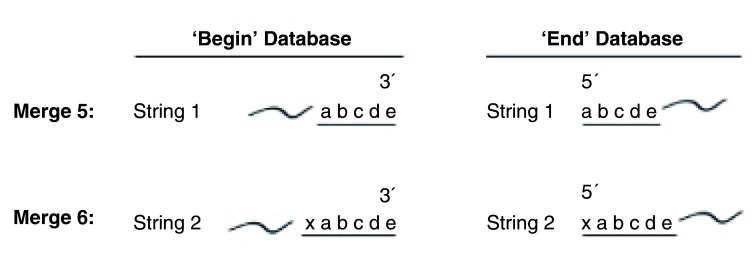
Pictoral depiction of ‘Begin’ and ‘End’ database. The contents of the ‘Begin’ and ‘End’ databases are represented along with the shared amino acid residues at the 3’ and 5’ ends, respectively. The ‘merge5’ command identified the String1 pair in the ‘Begin’ and ‘End’ databases due to the shared sequence ‘abcde’. The ‘merge5’ command failed to identify the String2 pair since six residues are shared. The ‘merge6’ command, however, will recognize String2 in the ‘Begin’ and ‘End’ databases, but would fail to recognize pairs that shared seven or more residues at the 3’ and 5’ ‘End’.

Algorithm 1 describes the process of merging transcripts ( SI Figure 1). For a given length (which varies from 5 to 15 in this case), the 5’ and 3’ sequences and identifiers of each transcript are stored in new string databases: 3’=Begin; 5’=End. Repetitive strings (strings that have only two letters) are ignored, as it is difficult to ensure their uniqueness. For each string of *n* length in the Begin (3’) string database, we find whether: a) unique matches of *n* length (one-to-one mapping) are present in the End (5’) string database and b) that the prefixes (initial transcript identifiers) of the transcripts are the same.

Algorithm 1. **MergeTRS - Merge two transcripts**

**Input:** *φ _TRS_* ⇐ Set of transcripts

**Output:** *φ _TRSMERGED_*: Pairs of transcripts that can be

             merged

**begin**

      
*φ _TRSMERGED_* ← 0;

      
**while** *NewStatesAdded* **do**

           
**foreach** *TRS _i_ in φ _TRS_* **do**

                 
*φ _BEGIN_* ← 0;

                 
*φ _END_* ← 0;

                 
**foreach** *len:5..15* **do**

                      AddBeginingofTRS( *φ _BEGIN_*, *TRS _i_*, *len*);

                      AddEndofTRS( *φ _END_*, *TRS _i_*, *len*);

                 
**end**

                 
**foreach** *string _i_ in φ _BEGIN_* **do**

                      /* ignore strings that have less than 3 letters, these are repetitive*/

                      IgnoreRepeats( *string _i_*);

                      if(∃ only one *string _j_* in *φ _END_*) such that prefixof( *TRS _i_*) == prefixof( *TRS _j_*))[

                      
*φ _TRSMERGED_* ←

                      AddtoMergeableSet( *TRS _i_,TRS _j_*);

                      ]

                 
**end**

           
**end**

      
**end**

      return *φ _TRSMERGED_*;

**end**

Algorithm 2 describes the iterative method for identifying homologous genes in the genome based on the transcriptome. First, the transcriptome is converted to a set of protein sequences by choosing the appropriate ORF (described above) as the representative protein sequence, and a BLAST database (TRSDB) is created. An input protein sequence (possibly from another organism) of a gene of interest is used to query TRSDB using BLAST ^29^. This results in a set of significant transcript matches which is pruned based on a cutoff identity (40% in this case) and the criterion that the sequence length should not differ more than another parameterizable value (50 in this case). Both these transcripts are now potential genes, and the above mentioned process is repeated for each of them, until no new transcripts are added.

Algorithm 2. **FindGene - Iterative method to identify homologous genes based on the transcriptome**

**Input:** *G* ⇐ Amino acid sequence of gene

**Input:** *TRSDB* ⇐ BLAST database of the protein sequences from each transcript, choosing the longest ORF as the representative protein sequence

**Input:** *identitycutoff* ⇐ Ignore matches which are less than *identitycutoff* % identical to the sequence under consideration

**Input:** *lengthcutoff* ⇐ Ignore matches where the sequence length differs by more than *lengthcutoff* % from the sequence under consideration

**Output:** *φ _genes_*

**begin**

     
*φ _genes_* ← *G*;

     
*φ _processed_* ← 0;

     
*NewStatesAdded* ← 1;

     
**while** *NewStatesAdded* **do**

          
*NewStatesAdded* ← 0;

          
**foreach** *G _i_ in φ _genes_ such that G _i_ is not in*

          
*φ _processed_* **do**

               
*φ _processed_* ← *G _i_*;

              
$\phi_{i}^{\text{BLAST}}$ = BLAST *G _i_* on *TRSDB*;

               
**foreach** *TRS _i_ in* $\phi_{i}^{\text{BLAST}}$ **do**

                    
*difflength* ←

                    
*length*( *G _i_*) – *length*( *TRS _i_*) ;

                    if(identity( *TRS _i_*, *G _i_*) > *identitycutoff* ^

                    ( *difflength* < *lengthcutoff*)) [

                    
*NewStatesAdded* ← 1;

                    
*φ _genes_* ← *TRS _i_*;

                    ]

               
**end**

          
**end**

**     end**

     /* This is not a TRS, but an input - remove this from the set*/

     remove *G* from *φ _genes_*;

     return *φ _genes_*;

**end**

The raw counts for each transcript is normalized according to Equation 1, assuming a read length of 100.

$${\text{Score}}_{\text{normal}}=100*[{\text{Score}}_{\text{raw}}/(\text{Length}(\text{transcript}))];\;\;\;\;(1)$$

The sequence alignment was done using ClustalW ^31^. The alignment images were generated using SeaView ^32^.

The runtimes for most of the processing required in YeATS is a few hours on a simple 16 GB, 16-core machine, barring the search for homologies in the BLAST ‘nr’ database. This search can be significantly accelerated when the organism under investigation has well-annotated protein databases (as in the current case), much in lines of the newly introduced SMARTBLAST ( http://blast.ncbi.nlm.nih.gov/smartblast/), to runtimes under a day.

### *In vitro* methods

Total RNA was isolated from the xylem region immediately external to the heartwood of a 16 year-old black walnut. The tree was felled in November, cross sections about 1 inch thick were taken from the base and dropped immediately into liquid nitrogen. After the sections were fully frozen they were transported to the lab on dry ice. The transition zone was then chiseled and the xylem was ground using a freezer mill. The RNA was extracted from 100g of ground wood using lithium chloride extraction buffer, and subsequently treated with DNAse (to remove genomic DNA) using an RNA/DNA Mini Kit (Qiagen, Valencia, CA) per the manufacturers protocol. Presence of RNA was confirmed by running an aliquot on an Experion Automated Electrophoresis System (Bio-Rad Laboratories, Hercules, CA).

The cDNA libraries were constructed following the Illumina mRNA-sequencing sample preparation protocol (Illumina Inc., San Diego, CA). Final elution was performed with 16 *μ*L RNase-free water. Each library was run as an independent lane on a Genome Analyzer II (Illumina, San Diego, CA) to generate paired-end sequences of 85bp in length from each cDNA library.

Prior to assembly, all reads underwent quality control for paired-end reads and trimming using Sickle ^33^. The minimum read length was 45bp with a minimum Sanger quality score of 35. The quality controlled reads of 19 libraries from *J. nigra* were *de novo* assembled with Trinity v2.0.6 ^14^ (standard parameters with minimum contig length of 300bp) (manuscript in submission, bioproject id PRJNA232394). Subsequently, the reads from the TZ from *J. nigra* was aligned to this transcriptome and counts obtained by BWA’s short read aligner v.0.6.2 (‘bwa aln’) ( http://bio-bwa.sourceforge.net/) ^34^. The Illumina reads for the transition wood transcriptome can be accessed at http://www.ncbi.nlm.nih.gov/sra/SRX404331.

## Results

**Figure 2.**
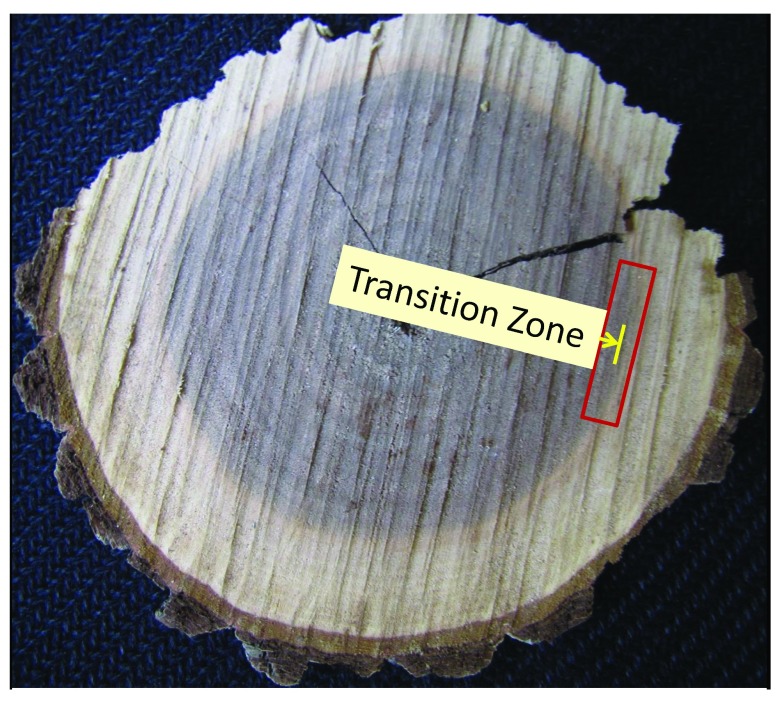
Heartwood/sapwood transition zone in black walnut. A cross section of a mature black walnut ( *Juglans nigra*) stem showing the light-colored sapwood (Secondary xylem), darkly colored heartwood which contains no living cells. The transition zone (TZ) is immediately external to the heartwood highlighted by the yellow line in the red box. Cell death is actively occurring in this TZ tissue.

The input dataset to the YeATS tool was a set of transcripts, transcript identifiers and their corresponding raw counts (see Supporting information), obtained from the tissue at the heartwood/sapwood transition zone (TZ) in black walnut ( *Juglans nigra* L.) ( Figure 2). These raw counts were normalized (see Methods), and transcripts with zero counts were ignored (see rawcounts.normalized.TZ in Dataset 1). There were ~24K such transcripts $(\phi_{\text{transcript}}^{\text{TZ}}).$

YeATS Dataset README
FASTADIR.tgz : 24k transcripts
ORFS.tgz : open reading frames from 24k transcripts computed from the ‘getorf’ tool from the Emboss suite.
list.merged.txt : transcripts that have been merged based on overlapping ends
High.TZ.genome.annotated.csv : transcripts having only one ORF with a high significance match
Lower.TZ.genome.annotated.csv : transcripts having only one ORF with a lower significance match
TZ.genome.annotated.none.csv : transcripts with no match
TZ.genome.errors : transcripts which have two ORFs matching with high significance to the same gene
TZ.genome.annotated.morethanone.csv : transcripts having more than one ORFs which match to different genes with high significance
rawcounts.TZ: Raw counts
rawcounts.normalized.TZ: Normalized counts Click here for additional data file. Copyright: © 2015 Chakraborty S et al. 2015 Data associated with the article are available under the terms of the Creative Commons Zero "No rights reserved" data waiver (CC0 1.0 Public domain dedication).

In order to associate a transcript to a specific open reading frame (ORF), the ORFs of $\phi_{\text{transcript}}^{\text{TZ}}$ is obtained using ‘getorf’ from the Emboss suite ^30^ (see ORFS.tgz in Supporting information) ( Figure 1). The three longest ORFs for each transcript is BLAST’ed to the full non-redundant protein sequences (‘nr’) database, and the results were used to characterize the genes.

There were ~1200 transcripts that had possible sequencing or assembly errors, ~22K transcripts that had significant matches (E-value<E-12) in the ‘nr’ database, 113 transcripts that had lower matches (E-12<E-value<E-08) in the ‘nr’ database, ~700 transcripts that had no matches in the ‘nr’ database and about 200 transcripts that could be merged based on overlapping amino acid sequences. We describe these in detail below.

### Possible sequencing error or mis-assembly of transcripts

**Figure 3.**
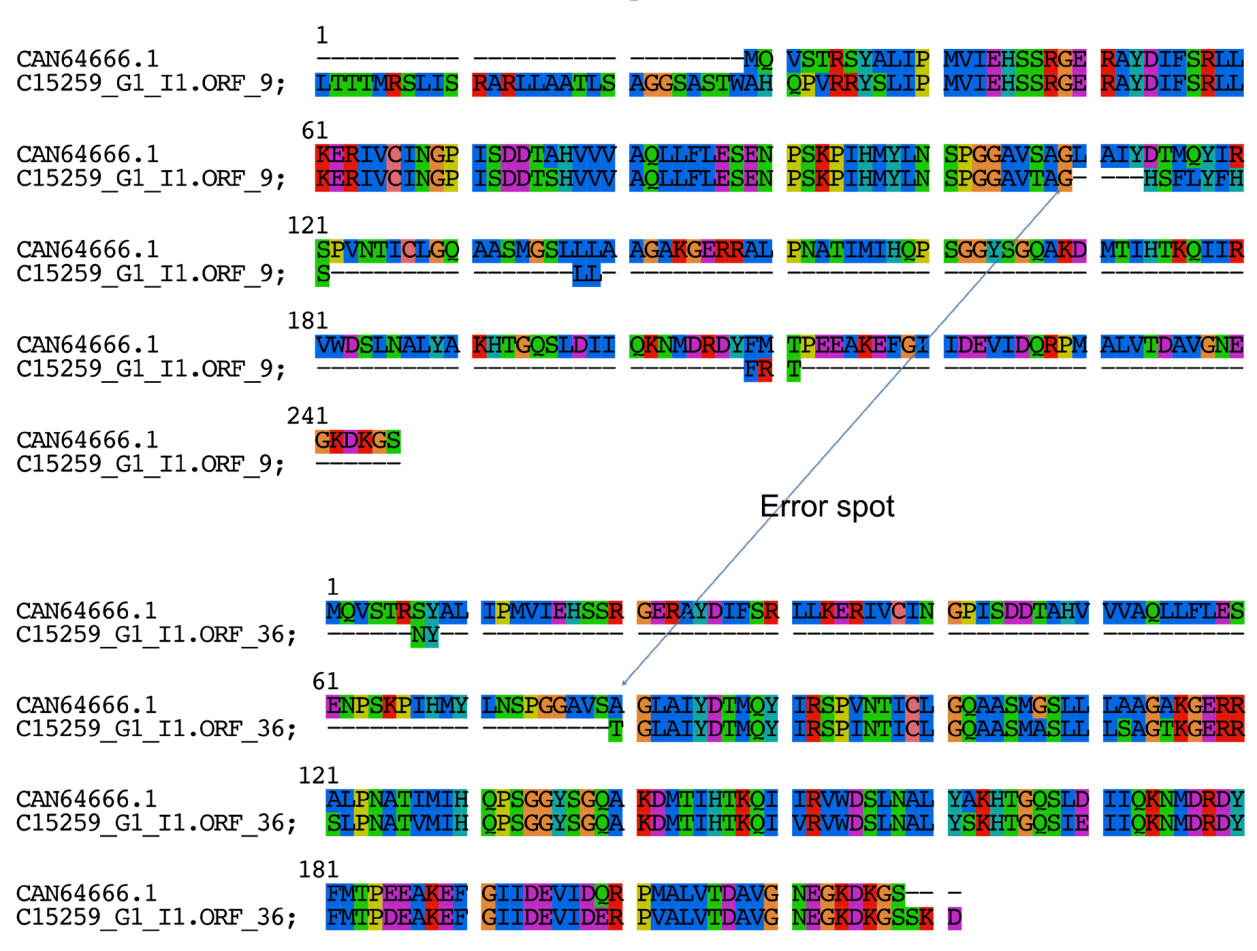
Error detection in sequencing or transcript assembly by YeATS. Transcript C15259_G1_I1 has two ORFs - 9 and 36 - both of which match to the mitochondrial ATP-dependent Clp protease proteolytic subunit 2, mitochondrial (GenBank: CAN64666.1) from *Vitis vinifera* with E-values of 6E-92 and 7E-45, respectively. It is likely that the error occurred near the amino acid sequence ‘SAG’ marked in the figure. The current transcriptome of the walnut TZ had a 5% (1200 out of 24,000) error rate for this class of error.

We observed transcripts that had multiple ORFs that matched to the same gene with high significance (E-value<E-10). The possibility that such an occurrence is not an experimental artifact is low. Transcript C15259_G1_I1 is one such example, having two ORFs - ORF_36 (length = 144) and ORF_9 (length = 122), both of which match to the mitochondrial ATP-dependent Clp protease proteolytic subunit 2 ^35^ (GenBank: CAN64666.1) from *Vitis vinifera* with E-values of 6E-92 and 7E-45, respectively. Figure 3 shows the alignment of these two ORFs to the *Vitis vinifera* protein indicated the possible site of the sequencing error or transcript misassembly. This aspect of the YeATS methodology can be used to estimate the sequencing and transcript assembly error rate. For example, in the current transcriptome of the walnut TZ, we found a 5% (1200 out of 24,000) error rate.

### Long repeat within the same transcript

**Figure 4.**
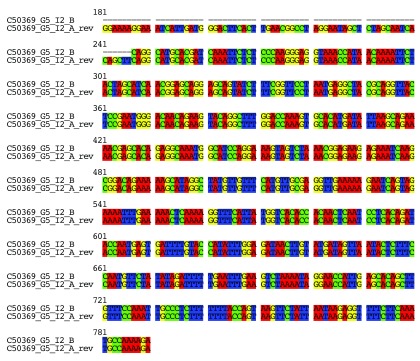
Erroneous transcripts with an exact long repeat (on the reverse strand). Transcript C50369_G5_I2 had an ORF (length = 143, Uniprot id: XP_009362671, uncharacterized protein), with an exact match on the reverse strand. There were only eight such cases, and they could be manually corrected.

A small number of transcripts had long repeats (on the reverse strand), as identified by transcripts that had multiple identical ORFs. For example, transcript C50369_G5_I2 has two ORFs (length = 143) that matched to an uncharacterized protein (Uniprot id: XP_009362671, E-value= 4e-13). These ORFs were located on the reverse strand, and were exactly the same ( Figure 4). There were only 8 such cases.

### Merging transcripts

**Figure 5.**
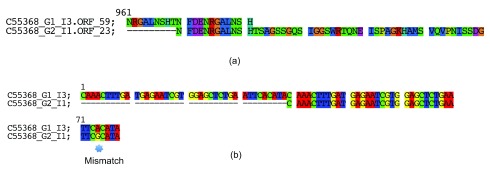
Transcripts that could be merged. ( **a**) Transcripts C55368_G1_I3 and C55368_G2_I1 could be merged based on a stretch of 12 amino acids (NFDENRGALNSH) obtained from their ORFs. ( **b**) The partial nucleotide sequences of these transcripts shows the repeat with only a single nucleotide difference. The indicated single nucleotide difference may explain the failure of the assembly program to merge these two transcripts. Interestingly, the transcript C55368_G1_I3 had two exact repeats of this stretch at the end which may have contributed to the failure of the assembly program to merge these transcripts.

About ~200 transcripts have been merged using conservative metrics by YeATS (see Methods, list.merge in Supporting information). For example, transcripts C55368_G1_I3 and C55368_G2_I1 were merged based on a stretch of 12 amino acids (NFDENRGALNSH) ( Figure 5). The indicated single nucleotide difference might be the reason for the failure of the assembly program to merge these two transcripts. Transcript C55368_G1_I3 had two exact repeats of this stretch, which is a likely assembly error.

**Figure 6.**
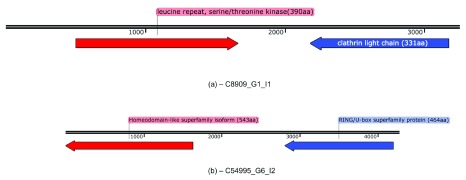
Identification of transcripts encoding multiple genes. These ORFs belong to the same transcript, and have significant matches to different proteins. ( **a**) Genes on the reverse strand, having no overlap - clathrin light chain (value=3E-126) and a leucine repeat rich receptor-like serine/threonine protein kinase (E-value=0). ( **b**) Genes on the same strand, having no overlap - RING/U-box superfamily protein (E-value=7E-149) and a homeodomain-like superfamily protein isoform (E-value=0).

### Single transcripts with two ORFs

Some transcripts were associated with multiple ORFs with distinct significant matches in the ‘nr’ database. We demonstrate this for the transcript C8909_G1_I1, which had two ORFs - ORF_104 (length = 331) and ORF_45 (length = 390) which matched to a clathrin light chain ^36^ (Uniprot id:XP_006481016.1, E-value=3E-126) and a leucine repeat rich receptor-like serine/threonine protein kinase ^37^ (Uniprot id: XP_007026739.1, E-value=0), respectively. These ORFs were on opposite strands, and did not overlap. It was not possible to ascertain which was the correct gene product, and it is a distinct possibility that both strands were transcribed ^38^. A slightly different situation arose when both the ORFs were on the same strand ^39^, as in the case of the transcript C54995_G6_I2. For example, in transcript C54995_G6_I2, there were two ORFs - ORF_157 (length = 464) and ORF_231 (length = 543) that matched to a RING/U-box superfamily protein ^40^ (Uniprot id: XP_007042454.1, E-value=7E-149) and a homeodomain-like superfamily protein isoform ^41^ (Uniprot id: XP_007030696.1, E-value=0), respectively. Both of these proteins were on the same (reverse) strand of the transcript. These transcripts are candidates for chimeric ^42^ or fusion ^43^ genes, since the ribosome is known to bypass small nucleotide stretches separating two ORFs ^44^.

### Highly transcribed genes

Table 1 shows the transcripts with the highest counts. Interestingly, the most abundant transcript had no homologous counterpart in the full BLAST ‘nr’ or ‘nt’ database (GenBank accession: C52369_G2_I1). A proline-rich protein (PRP), a part of the protein superfamily of cell wall proteins consisting of extensins and nodulins, was found to have the second most abundant transcript ^23, 45^. Proline comprises 19% of the amino acids in the ORF of this transcript. PRPs are found as structural proteins in wood, and it was hypothesized that these proteins occur in the xylem cell walls during ligniflication, and influence the properties of wood ^46^. PRPs were associated with carrot storage root formation ^47^, were wound and auxin inducible ^47^ and implicated in cell elongation ^48^. PRPs are also an integral component of saliva responsible for the precipitation of antinutritive and toxic polyphenols by forming complexes ^49^. Two DNAJ/HSP40 chaperone proteins, which are involved in proper protein folding, transport and stress response, showed high transcriptional levels ^28^. Two DNAJ/HSP40 chaperone homologs (GenBank accession id: BI677935 and BI642398) were shown to be differentially expressed during summer at the sapwood/heartwood TZ of black locust ^50^. The transcription levels of dehydrin-related proteins were shown to be seasonally regulated in the wood of deciduous trees ^26, 51^. However, this dehydrin protein is homologous to a 24kDa dehydrin (Uniprot id: AGC51777) from *Jatropha manihot*, a drought resistant plant ^52^, unlike the ~100kDa proteins investigated in 26. Senescence-associated proteins, and the related tetraspanins, were also highly transcribed ^27^. One highly expressed transcript was homologous to a protein that is yet to be characterized.

**Table 1.** A sample of highly transcribed genes with high normalized counts (NC). There are several highly transcribed genes in the representative sample of the transcriptome from the tissue at the heartwood/sapwood transition zone (TZ) in black walnut that did not have any significant homologs (NSL) in the complete ‘nr’ or ‘nt’ database. For the ‘nr’ database, we use the three longest ORFs as query. The significance of dehydrins, senescence-associated and DNAJ proteins can be observed through their transcription abundance.

ID	NC	Description	E-value
C52369_G2_I1	43040	NSL (putative extensin based on amino acid composition)	-
C51134_G2_I2	15200	ref|XP_008224364.1|PREDICTED: extensin-like [ *Prunus mume*]	1e-08
C40830_G1_I1	14169	ref|XP_006365673.1|dnaJ protein homolog isoform X2 [ *Solanum tuberosum*]	0
C46581_G1_I1	10651	PREDICTED: Probable zinc transporter protein [ *Phoenix dactylifera*]	8e-09
C51134_G2_I3	10631	emb|CAN59948.1|hypothetical protein VITISV_043422 [ *Vitis vinifera*]	6e-09
C44353_G2_I1	7769	gb|AGC51777.1|dehydrin protein [ *Manihot esculenta*]	6e-09
C44353_G1_I1	6652	gb|AAF01465.2|AF190474_1 bdn1 [ *Paraboea crassifolia*]	2e-19
C43130_G3_I1	6601	gb|KEH16988.1 |j senescence-associated protein, putative [ *Medicago truncatula*]	2e-129
C44922_G1_I1	5584	ref|XP_008363477.1|tetraspanin-3-like [ *Malus domestica*]	2e-169
C40830_G1_I2	5113	ref|XP_007010484.1|DNAJ [ *Theobroma cacao*]	0

### Finding genes

**Figure 7.**
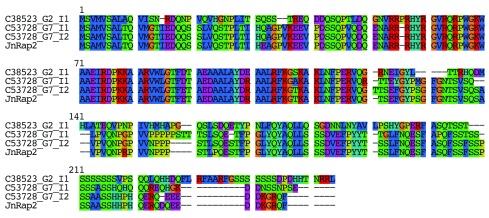
Finding genes from a template sequence. Multiple sequence alignment of possible genes for a transcription factor that had a AP2 DNA binding motif compared to JnRap2, which was deduced from an EST sequence obtained from tissue at the heartwood/sapwood transition zone in black walnut.

We demonstrated the (iterative) gene finding methodology in YeATS on a transcription factor that has an AP2 DNA binding motif (RAP2.6L in *Arabidopsis*, At5g13330) ^53^. This protein showed differential tissue specific expression, and is likely to be involved in plant developmental processes and stress response ^54^. Recently, the sequence of a homolog of RAP2.6L was deduced (Uniprot id: C1KH72, JnRap2) from an EST sequence isolated from tissue at the heartwood/sapwood TZ in black walnut ( *Juglans nigra* L.), and its role in the integration of ethylene and jasmonate signals in the xylem and other tissues was established ^55, 56^. Using the sequence of JnRap2, we probed for other RAP2 genes in the TZ of walnut. We found three possible genes (C38523_G2_I1, C53728_G7_I1 and C53728_G7_I2) ( Figure 7). It was observed that C53728_G7_I2 was closest to the JnRap2 gene (97.4% identity, 98.2% similar), and is probably the same gene. C53728_G2_I1 was also significantly homologous to the JnRap2 gene (84.4% identity, 92.4% similar), and it appears to be an allelic or splice variant, a conflict that can be resolved after the publication of the complete walnut genome. Raw counts (see Supporting information) demonstrated that the transcript C38523_G2_I1 had negligible expression levels in TZ, corroborating the previous detection of only one RAP2 protein in 55.

### Transcripts with no significant matches in the ‘nr’ database - possible long non-coding RNA genes?

The top three ORFs of ~600 transcripts had no match in the BLAST ‘nr’ database. Although these may be unique genes, another possibility that must be considered is that these are non-coding RNA genes ^2^. The nucleotide sequences of these 600 transcripts were BLAST’ed to the database of noncoding RNAs in *Arabidopsis* ^22^. Three matches were identified: C52424_G5_I11, C52424_G5_I4 and C53565_G3_I1. Both C52424_G5_I11 and C52424_G5_I4 are homologous to CR20, a cytokinin-repressed gene in excised cotyledons of cucumber, hypothesized to be non-coding RNA ^57^. Analogous to the current work, the CR20 gene had alternate splicing ^57^. C53565_G3_I1 had a 100% match to the *Arabidopsis* locus ATMG01380, a mitochondrial 5S ribosomal RNA, which is a component of the 50S large subunit of mitochondrial ribosome ^58^.

## Discussion

High-throughput mRNA sequencing (RNA-Seq) has revolutionized the field of transcript discovery, providing several advantages over traditional methods ^7, 8^. Following isolation and fragmentation of RNA and subsequent generation of cDNA libraries, a high-throughput sequencing platform is selected to generate short reads ^59^. Reconstruction of transcripts from these short reads (assembly) may be performed using a reference genome or *de novo* algorithms ^15– 18, 21, 60^. Sequencing biases, variable coverage, sequencing errors, alternate splicing and repeat sequences are some of the challenges faced by these assemblers ^14, 61^.

Several post assembly computational tools provide further curation of transcripts resulting from the assemblers. The curation step involves identifying redundancies ^19, 20^, finding coding regions ^62^, annotating the transcripts ( https://transdecoder.github.io/) and detecting inaccuracies by aligning the transcripts to the genome ^63^. In the current work, we present an integrated workflow for RNA-seq analysis (YeATS). YeATS includes most features of the tools mentioned above. Additionally, YeATS delivers several capabilities absent in these tools. A comprehensive BLAST analysis of the top three open reading frames of each transcript enables the identification of erroneous transcripts arising out of sequencing or assembly errors. These erroneous transcripts can be classified as: a) transcripts that have not been merged, b) transcripts that result in broken ORFs and c) transcripts that have long improbable repeats. Finally, YeATS provides annotation of the genes, enumerates homologous genes based on a template sequence and specified similarity threshold and identifies transcripts with multiple ORFs. The ribosome is known to bypass small nucleotide stretches separating two ORFs ^44^. These are rare events, however, and thus unlikely to apply to the ~1200 transcripts that have broken ORFs pointing to the same gene ^64^. Transcripts having multiple ORFs on the same strand are good candidates for chimeric ^42^ or fusion ^43^ genes dependent on ribosome bypassing.

**Figure 8.**
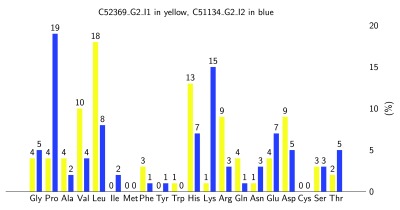
Percentage amino acid composition of the two most highly transcribed genes. C52369_G2_I1 has a high percentage of leucine, histidine and valine, and is a putative extensin. C51134_G2_I2 is proline and lysine rich, and is homologous to an extensin and nodulin.

The current work reveals and corroborates several aspects of the biology of hardwood trees. Probably, the most interesting is the detection of a highly transcribed gene (C52369_G2_I1) with no known homologs in the complete protein and nucleotide BLAST database, or significant matches in a database of long non-coding RNA genes ^22^. If indeed the longest ORF of this transcript encodes a protein, it is 143 amino acids long, and is leucine (18%), histidine (13%) and valine (10%) rich ( Figure 8). Although it is likely that this is a protein with leucine rich repeats, these proteins are typically larger proteins ^65^. On the other hand, histidine and valine rich extensins have been reported to be constituents of plant cell walls of dicots ^23^. The regulatory stimuli of extensins are different for monocots (which also have different amino acid composition) and dicots ^23^. A significant presence of extensin-like proteins in the cell wall of both developing and mature xylem (wood) have been reported for pine ^46, 66^. The publication of the walnut genome will aid the characterization of these genes by elucidating its promoter sequences.

Well characterized proteins like proline-rich proteins ^25, 46^, dehydrins ^26^, senescence-associated proteins ^27^ and DNAJ/HSP40 chaperone ^50^ proteins were also abundant in the transcriptome. While *Arabidopsis* supports secondary growth, it fails to accumulate wood; it is therefore interesting to identify highly transcribed genes that are missing in the *Arabidopsis* proteome ( Table 2). The DNAJ/HSP40 chaperone, dehydrins and tetraspanin proteins are found in the *Arabidopsis* proteome (TAIR10_pep_20101214 ^67^), while the putative extensin, the proline-rich protein, a probable zinc transporter protein, an uncharacterized protein and senescence-associated protein appear to be unique to the walnut proteome.

**Table 2.** Identifying highly transcribed genes that are not present in the *Arabidopsis* proteome. The wood quality of walnut and *Arabidopsis* are quite different. It is informative to identify genes (proteins) that are absent in *Arabidopsis*, since they are likely to be responsible for the differences. The DNAJ/HSP40 chaperone, dehydrins and tetraspanin proteins are found in the *Arabidopsis* proteome, while the putative extensin, the proline-rich protein, a probable zinc transporter protein, an uncharacterized protein and senescence-associated protein appear to be unique to the walnut proteome.

TRS	Arabidopsis Id	Description	E-value	Significant?
C52369_G2_I1	AT5G04990.1	SUN1, ATSUN1 | SAD1/UNC-84 domain protein	0.75	
C51134_G2_I2	AT3G18440.1	AtALMT9, ALMT9 | aluminum-activated malat	0.046	
C40830_G1_I1	AT5G22060.1	ATJ2, J2 | DNAJ homologue 2 | chr5:730379	0	Y
C46581_G1_I1	AT5G51930.1	Glucose-methanol-choline (GMC) oxidore	8.1	
C51134_G2_I3	AT1G79090.2	FUNCTIONS IN: molecular function unkno	1.3	
C44353_G2_I1	AT1G76180.2	ERD14 Dehydrin family protein | chr1:28	1e-05	Y
C44353_G1_I1	AT1G20450.2	LTI29, LTI45, ERD10 | Dehydrin family pro	1e-07	Y
C43130_G3_I1	AT1G72110.1	O-acyltransferase (WSD1-like) family p	1.7	
C44922_G1_I1	AT3G45600.1	TET3 | tetraspanin3 | chr3:16733973–16735	8e-156	Y
C40830_G1_I2	AT3G44110.1	ATJ3, ATJ | DNAJ homologue 3 | chr3:15869	1e-179	Y

Also, we corroborated the presence of a transcription factor that has a AP2 DNA binding motif ^53, 55^, and identify additional splice/allelic variants with similar transcriptional levels. Once again, the knowledge of the walnut genome would enable a more profound understanding of such genes.

## Conclusions

In summary, the current work elucidates an integrated workflow for RNA-seq analysis with several innovative features for identifying and correcting erroneously assembled transcripts. We demonstrated this workflow by characterizing the transcriptome of the tissue at the heartwood /sapwood TZ in black walnut.

## Data availability

The data referenced by this article are under copyright with the following copyright statement: Copyright: © 2015 Chakraborty S et al.

Data associated with the article are available under the terms of the Creative Commons Zero "No rights reserved" data waiver (CC0 1.0 Public domain dedication). 

F1000Research: Dataset 1. YeATS Dataset, 10.5256/f1000research.6617.d49730 ^68^

## Software availability

### Latest source code

https://github.com/sanchak/YEATSCODE2

### Archived source code as at the time of publication

http://dx.doi.org/10.5281/zenodo.33137

### Software license

GNU General Public License version 3.0 (GPLv3)
