# Supplementary material for: YeATS - a tool suite for analyzing RNA-seq derived transcriptome identifies a highly transcribed putative extensin in heartwood/sapwood transition zone in black walnut
Source: F1000Res. 2015 Nov 6;4:155. Originally published 2015 Jun 17. [Version 2] doi: 10.12688/f1000research.6617.2 (PMC4732554; doi:10.12688/f1000research.6617.2)
Supplement: Peer review history

# Peer review: Referee response for version 2

**DOI:** 10.5256/f1000research.7788.r12065

**Published:** 2016-02-19

**Authors:** Binay Panda

**Affiliations:** 1 Genomics Applications and Informatics Technology laboratories (GANIT Labs), Bio-IT Centre, Institute of Bioinformatics and Applied Biotechnology(IBAB), Bangalore, Karnataka, India

Chakraborty et al. implemented a workflow for error estimation and correction, functional annotation and abundance estimation in RNA-seq data. They explored a methodology of analyzing longest ORFs of transcripts, using BLAST, as means to identify important genes. Although BLAST has been very commonly used for annotation, the authors proposed a very systematic approach of dividing the annotations into four sets based on the quality of the ORFs and their functional assignments.

**Overall comments:**

The authors have done a good deal of work in exploring an important topic. The article gives many ideas worth exploring and directions for annotating data from *de novo* transcriptome sequencing. However, I suggest that the authors pay special attention to the usage of different terms (genome, transcriptome, RNA-seq, reads etc.) and be consistent in their usage throughout the manuscript. Additionally, the figure legends need complete re-writing and the perl scripts need to be included in the supporting information. Several explanations need to be provided throughout the manuscript (details below).

A single round of proofreading will hugely improve the manuscript.

**Specific suggestions/questions:**

The title states that YeATS identifies a highly transcribed putative extension. This is a bit misleading. YeATS takes as input already assembled transcripts from Trinity, estimates their expression, employs BLAST to try and assign a function to the most highly transcribed gene, and finds no known homologs. Only from the manual examination of the amino acid content of its longest ORF do the authors come to the conclusion that it is a putative extension. It will perhaps be better to mention the error-detection and estimation capabilities of YeATS as its strongest points.

Introduction: What evidence is there to suggest that a putative protein with a high percentage of leucine, histidine and valine is a probable extension?

Where does Algorithm 1 fit in? Why is it needed? Merging of transcripts is mentioned for the first time in methods, without an explanation of why it is important and where are its potential applications? The authors should explain the algorithm and how it serves in detecting error in assembly/sequencing, and what kind of transcripts should be used in merging.

Algo 1 - Why is the length range defined as 5 to 15? Is there an explanation behind the selection? Also, 5-15 is nucleotides or amino acids?

Algo 1 - Please clarify why 2-letter strings have been called a 'repetitive'? They should be ignored, agreeably, because they are too short to give any reliable results, but it is misleading to call them repetitive?

In algorithm 1, what is meant by 'prefix' of transcripts? If the workflow, YeATS, is so strictly dependent on the Trinity transcript headers, it needs to be clearly mentioned in the article.

Is 'TRS' equivalent to 'transcript'? Please include a list of abbreviations used in the manuscript at the beginning of the manuscript.

Is there a rationale behind running BLAST a second time, when from the very first BLAST run, which gives the best ORF selection, the gene functional annotation can also be obtained? This could make use of the Algorithm 2 and reduce the overall runtime.

Algo2 - The genome does not picture anywhere here, therefore, is misleading to say 'identifying homologous genes in the genome based on the transcriptome.'

Algo2 - is 'lengthcutoff' a % or number of nucleotides or amino acids? 'Input' says %, whereas in the algo it is simple difference.

Algo2 - 'Both these transcripts are now potential genes', which 2 transcripts are the authors talking about?

Equation 1 - Which raw counts are these? How are they obtained?

What is 'score' in equation 1?

Sequence alignment of what was done using ClustalW? What was it used for?

*In vitro* methods

The authors should list the 19 different samples whose cDNA libraries were sequenced. Were these combined and assembled as a single transcriptome? Is this used a reference for read alignment and counts estimation? If not, what is the reference for read count estimation?

All bases below quality score 35 were trimmed? That is a very stringent criterion. You would lose a lot of data if not a single base below 35 quality score is retained post-trimming. Why did the authors choose to use this?

TZ - please expand the abbreviation.

bwa aln gives aligned files, not counts. What was used to generate the raw counts? Also, bwa is not a splicing aware aligner. Authors should use Bowtie instead to do this, which may prove to be a better alternative.

All the figures need to be improved. In all figures, the sequence in question should be highlighted / boxed to make it easier for the reader to follow what is being talked about.

Figure 5 legend - 'shows the repeat'. which repeat? Authors should clearly mention that there are 2 contiguous repeats of the same 39aa sequence in the transcript C55368_G1_I3. Also, there are 2 different reasons mentioned why the assembler could not merge the 2 transcripts in question. Please clarify which is the case.

GitHub repository

The README file requires substantial work. Some of the commands are quite confusing, comments are not clear, and perl scripts are not to be found. The numerous manual steps make the tool virtually un-useable.

I have read this submission. I believe that I have an appropriate level of expertise to confirm that it is of an acceptable scientific standard.

# Peer review: Referee response for version 2

**DOI:** 10.5256/f1000research.7788.r12066

**Published:** 2016-01-28

**Authors:** Michael Love

**Affiliations:** 1 Department of Biostatistics, Harvard TH Chan School of Public Health, Boston, MA, USA

I do not have expertise in transcript assembly, but I can comment on the general readability and usability of the article and tool suite.

1. As with the report from Dr. Charoensawan, I was expecting that the tool suite would be more integrated and documented than the collection of Perl scripts on the Github page. Nevertheless, the set of examples shown in the article I think are useful at showing the kinds of errors which arise from transcript assembly and presumably it is easy to use the scripts to identify such examples. The README is currently very minimal for a tool suite / integrated workflow, looking more like a set of comments above code rather than proper documentation of a tool suite. I would recommend changing from README to README.md, using Github markdown e.g. enclosing the commands in backticks, re-writing the comments as full sentences/paragraphs, separating the different steps by sub-headings, etc. A little effort here will make the landing page much more appealing. Also the documentation on the Github page should provide detailed information on the expected inputs and outputs.
2. The colors in Figure 1 make the text a bit hard to read. As Figure 1 is often where many readers will go to understand what you are doing, you would benefit from making the colors lighter so the text is easier to read, and removing unnecessary shading, 3D effects, etc.

I have read this submission. I believe that I have an appropriate level of expertise to confirm that it is of an acceptable scientific standard.

# Response

**Published:** 2016-01-30

**Authors:** Sandeep Chakraborty

**Affiliations:** Tata Institute of Fundamental Research, India

We thank you for taking the time to review this paper, and for your comments.

As we have mentioned previously in response to Dr Charoensawan, this manuscript is not meant to be a software article. The primary reason for this is that the flow is not push-button. One simple goal of this paper was to highlight downstream checks that can correct and improve the results of a heuristic assembler like Trinity (which is bound to have certain limitations).

An enhanced version of the merging algorithm (which struck us later on) is to check whether the E-value of the merged transcript decreases when BLAST'ed as compared to the two transcripts being merged. This definitely would point to a non-merged assembly.

Also, most of the methods described here are reasonably simple to code.

In time, as we figure out how to automate the scripts better, we will certainly incorporate your suggestions.

We will revise the manuscript with a simpler and less distracting version of Figure 1.

# Peer review: Referee response for version 2

**DOI:** 10.5256/f1000research.7788.r11300

**Published:** 2016-01-04

**Authors:** Varodom Charoensawan

**Affiliations:** 1 Mahidol University, Bangkok, Thailand

The authors have addressed most of my previous comments.

However, I still have one reservation on the use of *Arabidopsis* "proteome" (instead of of publicly available transcriptomes) as a benchmark for walnut transcripts found, in the section "Identifying highly transcribed genes that are not present in the *Arabidopsis* proteome". It would be useful if the authors could clarify this.

I have read this submission. I believe that I have an appropriate level of expertise to confirm that it is of an acceptable scientific standard.

# Response

**Published:** 2016-01-05

**Authors:** Sandeep Chakraborty

**Affiliations:** Tata Institute of Fundamental Research, India

Dear Dr Charoensawan,

   We would like to thank you once again for critically reviewing, and accepting the revised version.

As for the Table 2, which mentions the "Identifying highly transcribed genes that are not present in the Arabidopsis proteom

e" that you have found inadequately explained, we would like to specify that

1) This set of transcripts were first chosen as they have high expression levels (Table 1).

This table already shows the homology of the ORF's of these transcripts to the BLAST 'nr' database (apart from the first transcript, all other have some degree of homologous counterparts).

2) Next, Arabidopsis was chosen on purpose since (as we mention in the text) "it fails to accumulate wood".

Our intention was to extricate differences in the transcripts which probably define the wood quality of walnut. Choosing other proteomes that included other wood generating plants would not suffice to find such transcripts.

We will try to rephrase this part to make it more lucid when we make another version (it would be too small a change for a new version, otherwise).

best wishes,

Sandeep

# Peer review: Referee response for version 1

**DOI:** 10.5256/f1000research.7105.r10335

**Published:** 2015-10-05

**Authors:** Varodom Charoensawan

**Affiliations:** 1 Mahidol University, Bangkok, Thailand

Chakraborty and coworkers proposed a new platform for analysing transcriptomic data from RNA-seq (YeATS -Yet Another Tool Suite for analyzing RNA-seq derived transcriptome). The key feature of the tool highlighted by the authors is error estimation and correction of assembled transcripts, which is performed by analysing ORFs predicted in each transcript and merging of transcripts. This error-filtering step is supposedly missing in most other existing tools to date. In addition, YeATS is able to perform other common RNA-seq analytic tasks, such as transcript abundance estimation.

From the point of view of a frequent user of NGS tools, rather than a developer, I can see that such a tool can be useful for improving transcript assembly and estimation, especially in organisms with no or poorly annotated genomes. However, there are a number of points that, to me, would improve the tool and the article, and it would be great if the authors could address/clarify. I would be happy to discuss this further if any of my comments are not clear.

- It would be nice to include a performance evaluation of this new platform against existing tools, or with vs’ without the transcript error correction step by YeATS. One way to do this might be to take an existing RNA-seq dataset from a well-annotated organism such as *Arabidopsis* as a gold standard, and perform transcript assembly-estimation with and without correction by YeATS, and compare this to the transcript estimation using genomic information (e.g. by mapping reads to annotated transcriptomes/genomes). Does YeATS indeed improve the coverage and specificity of transcript estimation, for instance?
- Along the same lines as the comment above, it would be useful if the authors could comment on the time and/or computing resources required to perform the correction step. Also, are the accuracy and computing resources dependent on the read lengths and/or sequencing platforms? (Or is it intended for Illumina reads as used in the example?).
- Both the source code of YeATS and the data set used to illustrate its usage have been deposited and described at the end of the article. However, to this reviewer’s understanding, there is a set of Perl scripts deposited to Github, but it is still not clear to me how the tool/workflow should be implemented. The README does not seem to describe this. Could the author point out if there is already a guideline or documentation on how to use or integrate YeATS into an existing NGS workflow, if that already exists?
- To my understanding, the input of YeATS is a set of assembled transcripts performed by other tools (e.g. Trinity). However, this step was not clearly described in the “ *in vitro* methods” section on Page 5. Instead, it seems the trimmed reads were directly aligned to *J. regia *transcriptome, which is somewhat confusing. Could you please clarify these?
- The authors described the genes as highly “transcribed” in walnut (according to RNA-seq from this study?) that are not present in Arabiodopsis “proteome”. I found these to be slightly disconnected.          

 

Minor comments:

- Figure 1: Is the “no” label between the boxes “Choose longest ORF” to “Gene annotation” necessary?
- Page 3, 2 ^nd^ column, line 12: modify the text to “often in distinct regions of the transcript..” for clarity?
- Page 5, 1 ^st^ column: There were ~24K “of” such transcripts
- Figure 6’s legend: These ORF”s”

I have read this submission. I believe that I have an appropriate level of expertise to confirm that it is of an acceptable scientific standard, however I have significant reservations, as outlined above.

# Response

**Published:** 2015-10-13

**Authors:** Sandeep Chakraborty

**Affiliations:** Tata Institute of Fundamental Research, India

We would like to thank you for taking the time to review this paper. Please find our responses below.

**Chakraborty and coworkers proposed a new platform for analysing  transcriptomic data from RNA-seq (YeATS -Yet Another Tool Suite for analyzing RNA-seq derived transcriptome). The key feature of the tool highlighted by the authors is error estimation and correction of assembled transcripts, which is performed by analysing ORFs predicted in each transcript and merging of transcripts. This error-filtering step is supposedly missing in most other existing tools to date. In addition, YeATS is able to perform other common RNA-seq analytic tasks, such as transcript abundance estimation. From the point of view of a frequent user of NGS tools, rather than a developer, I can see that such a tool can be useful for improving transcript assembly and estimation, especially in organisms with no or poorly annotated genomes.**

We appreciate your positive comments, and the possibility of value addition by the suggested methodology for existing NGS flows. We believe that this is the first attempt to associate the key information encoded by transcripts within ORFs to assess the accuracy of the assembly.

**However, there are a number of points that, to me, would improve the tool and the article, and it would be great if the authors could address/clarify. I would be happy to discuss this further if any of my comments are not clear. It would be nice to include a performance evaluation of this new platform against existing tools, or with vs without the transcript error correction step by YeATS. One way to do this might be to take an existing RNA-seq dataset from a well-annotated organism such as Arabidopsis as a gold standard, and perform transcript assembly-estimation with and without correction by YeATS, and compare this to the transcript estimation using genomic information (e.g. by mapping reads to annotated transcriptomes/genomes). Does YeATS indeed improve the coverage and specificity of transcript estimation, for instance?**

YeATS evaluates the accuracy of a transcriptome, but it is dependent on downstream tools (like MAKER) to use this for proper annotation of the genes. Thus, there are no existing tools that we could compare it with directly. A highly curated database like the Arabidopsis would not be a fair comparison, since it might have been annotated looking at several data points. However, we have extensively used the YeATS pipeline in processing the newly sequenced walnut genome (manuscript in review), and established erroneous assembly for several genes of interest. The transcriptome from several other tissues were included in the genome study. Interestingly, the 5% error estimate remained the same.

**Along the same lines as the comment above, it would be useful if the authors could comment on the time and/or computing resources required to perform the correction step. Also, are the accuracy and computing resources dependent on the read lengths and/or sequencing platforms? (Or is it intended for Illumina reads as used in the example?).**

The run times for most of the processing required in YeATS is a few hours on a 16 GB, 16-core machine, barring the search for homologies in the BLAST ’nr’ database, which can be time-intensive for a comprehensive search. This search can be significantly accelerated when the organism under investigation has well-annotated protein databases (as in the current case), much in lines of the newly introduced SMARTBLAST (http://blast.ncbi.nlm.nih.gov/smartblast/), to run times under a day. Run times are dependent on the number of transcripts only, since the input to YeATS is an assembled transcriptome from a tool like Trinity. We have included this information in the manuscript.

**Both the source code of YeATS and the data set used to illustrate its usage have been deposited and described at the end of the article. However, to this reviewers understanding, there is a set of Perl scripts deposited to Github, but it is still not clear to me how the tool/workflow should be implemented. The README does not seem to describe this. Could the author point out if there is already a guideline or documentation on how to use or integrate YeATS into an existing NGS workflow, if that already exists?**

We have provided a README that describes the step in the YeATS workflow. However, this is not a push-button methodology, and goes through several steps, each of which is dependent on the previous step. Also, we have used custom schedulers, and thus several steps need to be adjusted depending on available resources. For example, the number of parallel jobs and the time-step between each submission is controlled through a custom-script. Thus, we have provided the key algorithms in detail in the paper for any developer to easily replicate our results. Furthermore, we are enhancing several of the programs based on more sophisticated algorithms (like using kmers, compression of data, etc). A proper release of this software will require some more time, but this manuscript was not meant to be a software article.

**To my understanding, the input of YeATS is a set of assembled transcripts performed by other tools (e.g. Trinity). However, this step was not clearly described in the in vitro methods section on Page 5. Instead, it seems the trimmed reads were directly aligned to J. regia transcriptome, which is somewhat confusing. Could you please clarify these?**

The input of YeATS is indeed a set of assembled transcripts performed by other tools like Trinity. We have modified the methods section to clarify this.

**The authors described the genes as highly transcribed in walnut (according to RNA-seq from this study?) that are not present in Arabiodopsis proteome. I found these to be slightly disconnected.**

We agree that these results are slightly disconnected to the general narrative of this paper, which focuses on post-assembly methodologies to assess the accuracy of assembled transcripts. However, these are interesting results that emerge during the analysis of the transcriptome of the transition zone of walnut, which has been obtained for the first time. And thus, though this may be of interest to researchers in the field, there is too little data to spin-off another paper to publish these findings.

**Minor comments:**

**Figure 1: Is the no label between the boxes Choose longest ORF to Gene annotation necessary?**

We have changed the label ’no’ to ’unannotated’. Long ORFs that do not have have significant matches are probably uncharacterized genes, and the genome could be annotated accordingly (although the annotation of novel genes is another problem not addressed in the current paper).

**Page 3, 2nd column, line 12: modify the text to often in distinct regions of the transcript.. for clarity?**

We have clarified this: ‘The ORFs map to different fragments of the same protein. This points to an error in the sequencing or the assembly, which breaks down the contiguous ORF into two fragments.’

**Page 5, 1st column: There were 24K of such transcripts Figure 6s legend: These ORFs**

We have made these modifications. Once again, we are thankful for your insightful comments, and hope to have addressed your concerns.
